# Challenges and directions in analytical paleobiology

**DOI:** 10.1017/pab.2023.3

**Published:** 2023-02-27

**Authors:** Erin M. Dillon, Emma M. Dunne, Tom M. Womack, Miranta Kouvari, Ekaterina Larina, Jordan Ray Claytor, Angelina Ivkić, Mark Juhn, Pablo S. Milla Carmona, Selina Viktor Robson, Anwesha Saha, Jaime A. Villafaña, Michelle E. Zill

**Affiliations:** Department of Ecology, Evolution, and Marine Biology, University of California, Santa Barbara, California 93106, U.S.A.; Smithsonian Tropical Research Institute, Balboa, Republic of Panama; GeoZentrum Nordbayern, Friedrich-Alexander-Universität Erlangen-Nürnberg (FAU), 91054 Erlangen, Germany; School of Geography, Earth and Environmental Sciences, University of Birmingham, Edgbaston, Birmingham B15 2TT, United Kingdom; School of Geography, Environment and Earth Sciences, Victoria University of Wellington, P.O. Box 600, Wellington, New Zealand; Department of Earth Sciences, University College London, Gower Street, London WC1E 6BT, United Kingdom; Life Sciences Department, Natural History Museum, Cromwell Road, London SW7 5BD, United Kingdom; Jackson School of Geosciences, University of Texas, Austin, Texas 78712, U.S.A; Department of Biology, University of Washington, Seattle, Washington 98195, U.S.A; Burke Museum of Natural History and Culture, Seattle, Washington 98195, U.S.A; Department of Palaeontology, University of Vienna, Josef-Holaubek-Platz 2,1090 Vienna, Austria; Department of Ecology and Evolutionary Biology, University of California Los Angeles, Los Angeles, California 90095, U.S.A; Universidad de Buenos Aires, Facultad de Ciencias Exactas y Naturales, Departamento de Ciencias Geológicas, Buenos Aires C1428EGA, Argentina; Instituto de Estudios Andinos “Don Pablo Groeber” (IDEAN, UBA-CONICET), Buenos Aires C1428EGA, Argentina; Department of Biological Sciences, University of Calgary, Calgary, Alberta T2N 1N4, Canada; Institute of Palaeobiology, Polish Academy of Sciences, ul. Twarda 51/55, 00-818 Warsaw, Poland; Laboratory of Paleogenetics and Conservation Genetics, Centre of New Technologies (CeNT), University of Warsaw, S. Banacha 2c, 02-097 Warsaw, Poland; Department of Palaeontology, University of Vienna, Josef-Holaubek-Platz 2, 1090 Vienna, Austria; Centro de Investigación en Recursos Naturales y Sustentabilidad, Universidad Bernardo O ‘Higgins, Santiago 8370993, Chile; Department of Earth and Planetary Sciences, University of California Riverside, Riverside, California 92521, U.S.A

## Abstract

Over the last 50 years, access to new data and analytical tools has expanded the study of analytical paleobiology, contributing to innovative analyses of biodiversity dynamics over Earth’s history. Despite—or even spurred by—this growing availability of resources, analytical paleobiology faces deep-rooted obstacles that stem from the need for more equitable access to data and best practices to guide analyses of the fossil record. Recent progress has been accelerated by a collective push toward more collaborative, interdisciplinary, and open science, especially by early-career researchers. Here, we survey four challenges facing analytical paleobiology from an early-career perspective: (1) accounting for biases when interpreting the fossil record; (2) integrating fossil and modern biodiversity data; (3) building data science skills; and (4) increasing data accessibility and equity. We discuss recent efforts to address each challenge, highlight persisting barriers, and identify tools that have advanced analytical work. Given the inherent linkages between these challenges, we encourage discourse across disciplines to find common solutions. We also affirm the need for systemic changes that reevaluate how we conduct and share paleobiological research.

## Introduction

Paleobiological research practices are evolving. Advances in computational power, modeling, and databases have equipped paleobiologists with new tools to analyze the fossil record. These advances have given rise to analytical paleobiology as a research topic within paleontology. Analytical paleobiology comprises paleobiological research that uses analytical (primarily quantitative) methods, including database-driven analyses, meta-analyses, and primary data analyses (Signor and Gilinsky 1991). Although analytical methods have long been used in paleontology, analytical paleobiology crystallized in the 1970s and 1980s following pivotal computational work that examined past biodiversity dynamics (e.g., Valentine 1969; Raup 1972; Raup et al. 1973; Sepkoski et al. 1981; Raup and Sepkoski 1982). Since then, it has matured both by adapting methods from other disciplines and by developing new methods specific to analyzing the fossil record (Raup 1991; Liow and Nichols 2010; Silvestro et al. 2014; Alroy 2020; Warnock et al. 2020). Analytical paleobiology has now grown to touch most subfields within paleontology. For example, analytical tools have been used to document macroevolutionary patterns, evaluate the causes and consequences of ecosystem change, and predict biotic responses to the current biodiversity and climate crises (Condamine et al. 2013; Finnegan et al. 2015; Muscente et al. 2018; Yasuhara et al. 2020). The demand for workshops on these topics, such as the Analytical Paleobiology Workshop (https://www.cnidaria.nat.uni-erlangen.de/shortcourse/index.html) and Paleontological Society Short Courses at the Geological Society of America annual meeting (https://www.paleosoc.org/short-courses), indicates that this research frontier is set to grow.

Although analytical paleobiology has been firmly established as a research topic, it continues to face challenges related to data analysis, synthesis, and accessibility. Some of these challenges are long-standing (Seddon et al. 2014), while others have been recently illuminated or even amplified by analytical advances (Raja et al. 2022). In response, many paleobiologists—particularly early-career researchers—have advocated for more collaborative, interdisciplinary, and open science. Their willingness to embrace new research practices has already begun to permeate the broader paleontological community. However, the guidelines and community buy-in that are needed to standardize these practices are still developing. As both the challenges that face analytical paleobiology and our capacity to tackle them evolve, it can be productive to monitor progress and reflect on how this research topic might continue to mature.

As one of the most recent cohorts to graduate from the Analytical Paleobiology Workshop (2019), we present this synthetic survey to signpost obstacles in analytical paleobiology from an early-career perspective and map them onto emerging solutions. We outline four interconnected challenges (Table 1), highlight recent progress, and collate a list of tools that have pushed analytical paleobiology in new directions (Supplementary Tables 1,2). By surveying a wide range of topics, we aim to link disparate advances and provide readers with entry points for engagement with each challenge, while directing them to comprehensive discourse on each. We also echo calls for more consistent and equitable approaches to data production, synthesis, and sharing within analytical paleobiology.

### Challenge 1: Measuring Biodiversity across Space and Time

**Figure 1:**
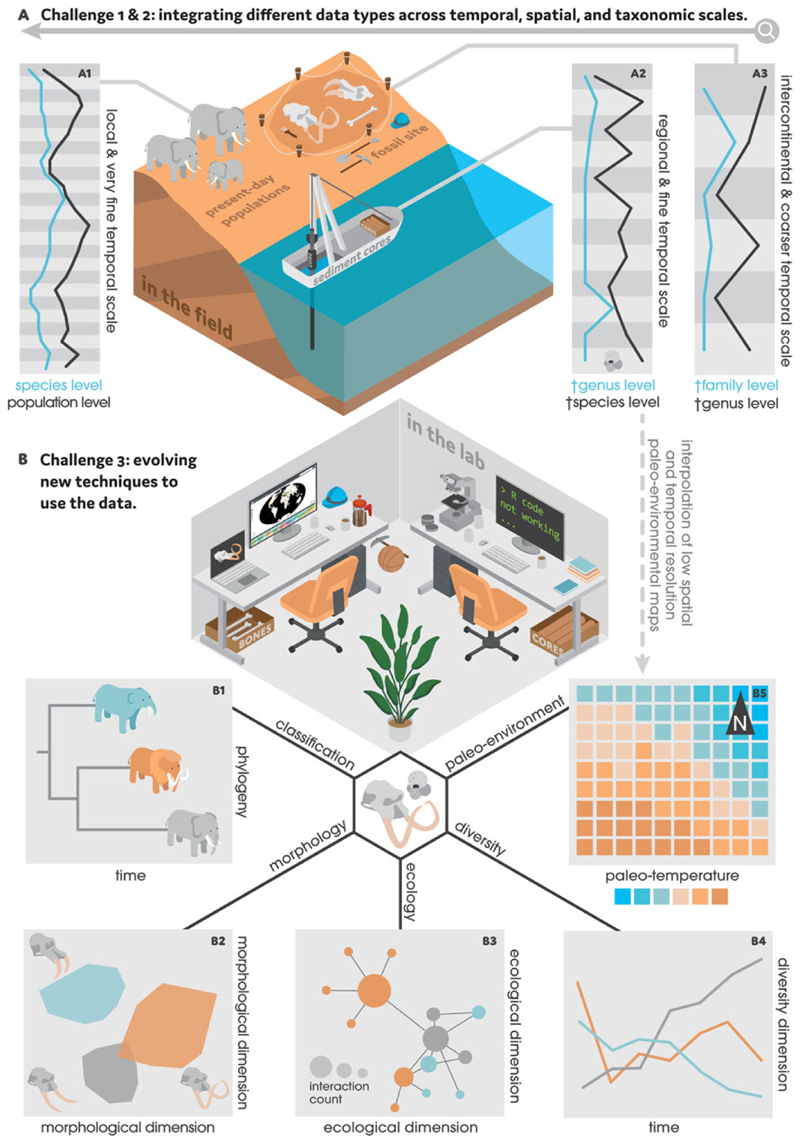
A, the interpretation and integration of different data types pose two major challenges in analytical paleobiology given their contrasting properties and scales. Moving from fine to coarse: A1, real-time monitoring data—indicated here by elephants—often having a very fine temporal (days, months), spatial (localities, sites), and taxonomic (populations, species) resolution; A2, microfossil data—often recovered from marine sediment cores and represented here by a *Globigerina* foraminifer fossil—having a fine temporal (thousands of years), spatial (basins), and taxonomic (species, genera) resolution; and A3, macrofossil data—indicated here by fossil remains from mammoth and *Deinotherium*—having a coarser temporal (millions of years), spatial (continents, worldwide), and taxonomic (genera, families) resolution. Microfossil, pollen, and geological data can also produce interpolated paleoenvironmental maps with low temporal (stages, periods) and spatial (km^2^) resolution (B5). B, to overcome these challenges, paleobiologists are developing quantitative approaches that use computer programming languages, software, and online databases. The scope of these analyses is vast, including but not limited to: B1, reconstructing phylogenetic relationships; B2, visualizing morphological differences among taxa; B3, quantifying biotic interactions (e.g., using ecological networks); B4, calculating diversity dynamics; and B5, pairing paleo-environmental patterns with taxon occurrences to model ecological niches through time.

The fossil record provides an invaluable but imperfect time capsule to explore how and why biodiversity has changed over Earth’s history. Early studies of deep-time biodiversity interpreted the fossil record at face value, but these interpretations are now widely documented to be confounded by a combination of geological, taphonomic, and sampling biases (Raup 1972, 1976; Sepkoski et al. 1981; Benton 1995; Smith and McGowan 2011; Walker et al. 2020). These biases can distort biodiversity estimates and hinder meaningful comparisons of fossil assemblages across space and time (Close et al. 2020a; Benson et al. 2021). In recent years, quantitative methods have accrued to alleviate some of these limitations, improving our ability to quantify true biodiversity patterns (Supplementary Table 2). However, researchers now face the challenge of creating transparent, reproducible workflows to navigate this landscape of resources as they prepare their raw data for analysis (Fig. 1). Here, we focus on four aspects of this workflow: taxonomic resolution, sampling standardization, spatial standardization, and time series analysis.

Estimates of taxonomic diversity are influenced by the resolution at which specimens are identified. Deep-time biodiversity patterns have long been quantified using counts of higher taxa, such as families (Sepkoski 1981; Labandeira and Sepkoski 1993) or genera (Sepkoski 1997; Alroy et al. 2008; Cleary et al. 2018). Genera are often preferred, because they are typically easier to identify, more robust to stratigraphic binning, and more taxonomically stable than fossil species (Allmon 1992; Foote 2000), such that they are considered to be a good substitute for biodiversity (Jablonski and Finarelli 2009). However, genera are not perfect proxies for species, which are more directly shaped by evolutionary and ecological processes (Hendricks et al. 2014). Nor are they immediately comparable with ecological data, which are often collected at the species level and are increasingly delineated using genetics (Pinzón et al. 2013; Zamani et al. 2022) (Fig. 1A). Authors have therefore called for greater transparency when analyzing genuslevel patterns (e.g., justifying the use of genera as well as reporting species-to-genus ratios) and discussing their implications for species (Hendricks et al. 2014). At the same time, the taxonomic work that underpins specimen identification remains chronically undervalued (Zeppelini et al. 2021; Gorneau et al. 2022; although see Costello et al. 2013). To preserve taxonomic knowledge, efforts could be made to invest in taxonomy courses (e.g., Smithsonian Training in Tropical Taxonomy), grants that fund curation and systematics (e.g., Paleontological Society Arthur James Boucot Research Grants), and taxonomy databases (Costello et al. 2013; Fawcett et al. 2022; Grenié et al. 2023). Investments in systematics might, in turn, encourage stronger connections between genus- and species-level analyses when studying biodiversity through time.

Biodiversity estimates are also sensitive to sampling. In the last two decades, numerous quantitative methods have been developed to compare numbers of taxa (taxonomic richness) among assemblages while accounting for variation in sampling. Yet there is still no one-size-fits-all approach, leaving researchers to weigh the trade-offs between different methods (Close et al. 2018; Alroy 2020; Roswell et al. 2021) or use multiple complementary methods (e.g., Allen et al. 2020). Richness estimators are a popular sampling standardization method (Alroy 2020). One example is shareholder quorum subsampling (Alroy et al. 2008; Alroy 2010a,b,c), which standardizes samples based on a measure of sample completeness, or coverage. This approach is mathematically similar to coverage-based rarefaction, which is commonly used in ecology to standardize samples when measuring species diversity (Chao and Jost 2012; Chao et al. 2020, 2021; Roswell et al. 2021). Other popular methods focus on macroevolutionary rates (e.g., origination and extinction). These range from relatively straight-forward equations (Kocsis et al. 2019) to more complex Bayesian frameworks (PyRate; Silvestro et al. 2014) and models that incorporate phylogenetic information (fossilized birth–death process; Heath et al. 2014; Warnock et al. 2020). Ecological methods, such as capture–mark-recapture (Liow and Nichols 2010), can also be used to infer biodiversity dynamics from incomplete samples but have not been as widely applied in paleobiology. The diversity of available methods underscores the complexity of measuring biodiversity but also presents an opportunity to establish best practices that fine-tune their usage. As consensus forms, paleobiologists and ecologists could collaborate to consolidate sampling standardization methods across disciplines (Challenge 2).

Although sampling standardization corrects for differences in sample completeness, it does not consider the geographic distribution of samples. Biodiversity patterns in the fossil record have traditionally been interpreted at global scales, yet these inferences are affected by the fossil record’s spatial structure (Bush and Bambach 2004; Vilhena and Smith 2013; Close et al. 2020b). If spatial variation in sampling is not addressed, apparent changes in biodiversity might reflect heterogeneity in depositional, environmental, or climatic conditions rather than genuine patterns (Shaw et al. 2020; Benson et al. 2021). Additionally, global analyses can mask local- or regional-scale variation in biodiversity (Benson et al. 2021). Researchers are increasingly using spatially explicit approaches to track biodiversity changes at nested spatial scales (Cantalapiedra et al. 2018; Womack et al. 2021). A variety of procedures have been developed in recent years to account for the spatial distribution of samples. Some are relatively simple metrics, such as the convex-hull area (Close et al. 2017) and number of occupied equal-area grid cells (Womack et al. 2021). Others are more complex, such as kernel density estimators (Chiarenza et al. 2019), summed minimum spanning tree length (Jones et al. 2021; Womack et al. 2021), and spatial subsampling procedures (Antell et al. 2020; Close et al. 2020b; Flannery-Sutherland et al. 2022). Some of the newer statistical approaches have been released with reproducible code or as R packages to allow updates from community members, providing an example of how methods in analytical paleobiology might mature (Challenge 3). Next steps could include efforts to establish incentive structures for contributing to this codebase, guidelines that compare methods, and workflows that link these packages.

Many paleobiological studies aim to quantify biodiversity through time, yet such analyses are complicated by variation in the fossil record’ s temporal resolution and quality (Fig. 1A). Because stratigraphic sequences are irregularly arranged in time and variably time-averaged, many common approaches to time series analysis (such as autoregressive integrated moving average, or ARIMA, models) cannot be readily applied (Kidwell and Holland 2002; Yasuhara et al. 2017; Simpson 2018; Fraser et al. 2021). Additionally, biodiversity dynamics can be scale dependent (Levin 1992; McKinney and Drake 2001; Lewan-dowska et al. 2020; Yasuhara et al. 2020) or can interact over different scales to yield emergent patterns (Mathes et al. 2021). Recent efforts to analyze biodiversity trends have been aided by advances in geochronology and age–depth modeling that provide more robust age control as well as models of depositional processes (Tomašových and Kidwell 2010; Kidwell 2015; Tomašových et al. 2016; Hohmann 2021; McKay et al. 2021). Progress has also been made by implementing analyses that can accommodate observations from different types of stratigraphic sequences while accounting for age-model uncertainty. In particular, generalized additive models (Simpson 2018), causal analyses like convergent cross mapping (Hannisdal and Liow 2018; Runge et al. 2019; Doi et al. 2021), multivariate rate-of-change analyses (Mottl et al. 2021), and machine learning methods (Karpatne et al. 2019) are changing research norms from describing temporal change to estimating statistical trends and making causal inferences among paleobiological time series. These approaches are still gaining momentum but will likely become more mainstream as they are incorporated into stratigraphic paleobiology and paleoecology training programs (Birks et al. 2012; Patzkowsky and Holland 2012; Holland and Loughney 2021).

As we highlighted earlier, paleobiological data often require extensive cleaning and standardization before they can be meaningfully analyzed. Open-source tools are being developed to streamline this workflow (e.g., Jones et al. 2022), typically in the R programming environment (Supplementary Table 2). Moving forward, this ecosystem of tools might encourage more reproducible data processing workflows within analytical paleobiology (Challenge 3). Nevertheless, quantitative methods cannot mitigate all biases, particularly those influencing the extent of the sampled fossil record. For example, variation in the preservational potential or environmental types represented by samples elude simple statistical corrections (Purnell et al. 2018; Walker et al. 2020; Benson et al. 2021; de Celis et al. 2021). Socioeconomic disparities can also exacerbate taphonomic or geological biases by fueling differences in sampling effort across countries (Amano and Sutherland 2013; Guerra et al. 2020; Moudrý and Devillers 2020; Raja et al. 2022) (Challenge 4). Although quantitative methods can help illuminate the potential severity of these biases, they cannot fill sampling gaps. As such, understanding the context in which samples were collected and communicating how they were interpreted will remain critical aspects of analytical paleobiology.

### Challenge 2: Integrating Fossil and Modern Biodiversity Data

Studies that link data from ancient and modern ecosystems offer holistic insight into processes spanning long timescales. For example, time series of taxon occurrences and environmental conditions in the fossil record can complement real-time monitoring to disentangle drivers of community assembly (Lyons et al. 2016), assess extinction risk (Raja et al. 2021), evaluate how ecosystems respond to disturbances (Buma et al. 2019; Tomašových et al. 2020; Dillon et al. 2021), and inform conservation decisions (Dietl et al. 2015; Kiessling et al. 2019). However, despite becoming more intertwined over the last decade, paleontology and ecology continue to progress as separate disciplines (Willis and Birks 2006; Goodenough and Webb 2022). Here, we outline four obstacles that impede the synthesis of paleobiological and ecological data, although these extend to other multiproxy work.

A first obstacle is data acquisition. Recent years have seen advances in data archiving as well as funding for projects that aggregate fossil and modern biodiversity data. Databases and museum collections, especially when digitized (Allmon et al. 2018), have promoted data discovery (Supplementary Table 1). In turn, application programming interfaces and web interfaces have facilitated data downloads. Examples include the paleobioDB R package, which extracts data from the Paleobiology Database (Varela et al. 2015), and the EarthLife Consortium (https://earthlifeconsortium.org), which queries the Paleobiology Database, Neotoma Paleoecology Database, and Strategic Environmental Archaeology Database (Uhen et al. 2021). As these tools have gained traction, there have been calls to standardize archiving and formatting protocols to increase database interoperability (Guralnick et al. 2007; Morrison et al. 2017; König et al. 2019; Wüest et al. 2020; Heberling et al. 2021; Nieto-Lugilde et al. 2021; Huang et al. 2022) as well as maintain interdisciplinary funding structures (e.g., Past Global Changes, https://pastglobal-changes.org) to ensure their future accessibility (Challenge 4).

A second obstacle stems from the practical aspects of integrating paleobiological and ecological data. Integrative analyses involve combining datasets with different units, scales, resolutions, biases, and uncertainties (e.g., paleoclimate proxies aligned with taxon occurrences; Fig. 1). These disparate data properties can hinder their inclusion in statistical models, which typically require consistent inputs that meet certain conditions (Yasuhara et al. 2017; Su and Croft 2018). In recent years, data synthesis has been streamlined by efforts to: (1) develop analyses that can accommodate heterogeneous datasets (Challenge 3); (2) calibrate complementary methods (Vellend et al. 2013; Buma et al. 2019); (3) standardize data harmonization protocols (König et al. 2019; Rapacciuolo and Blois 2019; Nieto-Lugilde et al. 2021); and (4) support interdisciplinary work (Ferretti et al. 2014). As integrative analyses become more common, best practices could be formalized to describe data properties, processing workflows, and boundaries of inference (e.g., Bennington et al. 2009; McClenachan et al. 2015; Wilke et al. 2016; Lendemer and Coyle 2021). One potential path forward is through frameworks that guide the practice of integration and provide conceptual scaffolding for new analytical techniques (Price and Schmitz 2016; Kliskey et al. 2017; Rapacciuolo and Blois 2019; Napier and Chipman 2022).

Conceptual barriers to data integration pose a third obstacle. These barriers often arise from differences between discipline histories, research goals, or methods (Szabó and Hédl 2011; Sievanen et al. 2012; Yasuhara et al. 2017). Process-, function-, or trait-based metrics offer a potential workaround. These metrics can help align datasets over multiple scales and identify common currencies that are grounded in ecological or evolutionary theory (Eronen et al. 2010; Ezard et al. 2011; Mouillot et al. 2013; Wolkovich et al. 2014; Yasuhara et al. 2016; Pimiento et al. 2017, 2020; Spalding and Hull 2021). This paradigm moves away from conventional attempts to explore an ecological or evolutionary process within the bounds of a single discipline, instead encouraging interaction among researchers who approach the same process from different angles. For example, resilience concepts from the ecological literature are already being applied to the fossil record (Davies et al. 2018; Scarponi et al. 2022). Moving forward, we echo existing calls to improve interdisciplinary communication (Benda et al. 2002; Boulton et al. 2005; Eigenbrode et al. 2007), which could help design meaningful metrics that are comparable between fossil and modern datasets.

Finally, the paleontological and ecological communities remain siloed despite their complementarity. They ask similar questions but use different terminology and tools over different timescales (Rull 2010). Interdisciplinary networks, conferences, departments, journals, and training programs can facilitate cross talk between these disciplines. Many examples already exist that provide blueprints for future partnerships. These include the Oceans Past Initiative (https://ocean-spast.org), Conservation Paleobiology Network (https://conservationpaleorcn.org), Crossing the Palaeontological-Ecological Gap meeting (https://www.cpegberlin.com) and journal issue (Dunhill and Liow 2018), and the PaleoSynthesis Project (https://www.paleo-synthesis.nat.fau.de). Collectively, such efforts could increase institutional support for interdisciplinary research and gradually change the culture of interdisciplinarity (Ferretti et al. 2014; Price and Schmitz 2016; Yasuhara et al. 2017). We could also learn from other interdisciplinary work such as social-ecological systems research, which links insights across the natural and social sciences (Schoon and van der Leeuw 2015). Ultimately, the high buy-in from early-career researchers in these initiatives bodes well for their longevity and impact.

### Challenge 3: Building Data Science Skills to Analyze the Fossil Record

Paleobiology is embracing “big data.” Not only are there more ways to collect high-resolution data (Olsen and Westneat 2015; del Carmen Gomez Cabrera et al. 2019; Goswami et al. 2019) and automate analyses using machine learning (Peters et al. 2014; Hsiang et al. 2018, 2019; Kopperud et al. 2019; Muñoz and Price 2019; Beaufort et al. 2022) but also new opportunities to tap into online databases (Alroy 2003; Brewer et al. 2012) (Fig. 1B). These advances have contributed to the volume, velocity, and variety of datasets that characterize big data (LaDeau et al. 2017). However, with this accumulating information (Supplementary Table 1) comes the need for more awareness of quantitative tools (Supplementary Table 2) and best practices for data analysis. Data science training programs paired with proactive efforts to collaborate with environmental data scientists could aid the transition toward more quantitative research.

There is a growing need for paleobiologists to learn statistical and coding skills. These skills are needed to analyze large heterogeneous datasets, implement reproducible coding practices (Nosek et al. 2015; Lowndes et al. 2017), and streamline analytical workflows (Wilson et al. 2017; Bryan 2018) (Challenges 1 and 2). Training could take the form of community-based discussions (Lowndes et al. 2019) and meetups (e.g., TidyTuesday), formal courses (e.g., Software Carpentry, https://software-carpentry.org), or independent instruction through coding tutorials (e.g., Coding Club, https://ourcodingclub.github.io/course.html). Additionally, data science topics could continue to be incorporated into paleobiology degree programs or taught as stand-alone analytical paleobiology courses. These training opportunities would provide a foundation for paleobiologists to use existing quantitative methods and create new software to analyze the fossil record.

As more paleobiologists run analyses in R, Python, and other coding languages, they could benefit from engagement with data scientists as well as with other disciplines that interface with data science, such as ecology and environmental science. Building computational skills might seem daunting, but there is no need to reinvent the wheel. Tools and infrastructure already exist (Sandve et al. 2013; Michener 2015; Hart et al. 2016; Lowndes et al. 2017; Wilson et al. 2017; Filazzola and Lortie 2022) that can be adapted to paleobiology (e.g., Barido-Sottani et al. 2020). Working groups at synthesis centers such as the National Center for Ecological Analysis and Synthesis (which produced the Paleobiology Database) and online communities like LinkedEarth (https://linked.earth) have already begun to foster data-driven collaborations in paleontology, foreshadowing how quantitative research agendas might progress.

### Challenge 4: Increasing Data Accessibility and Equity

**Figure 2:**
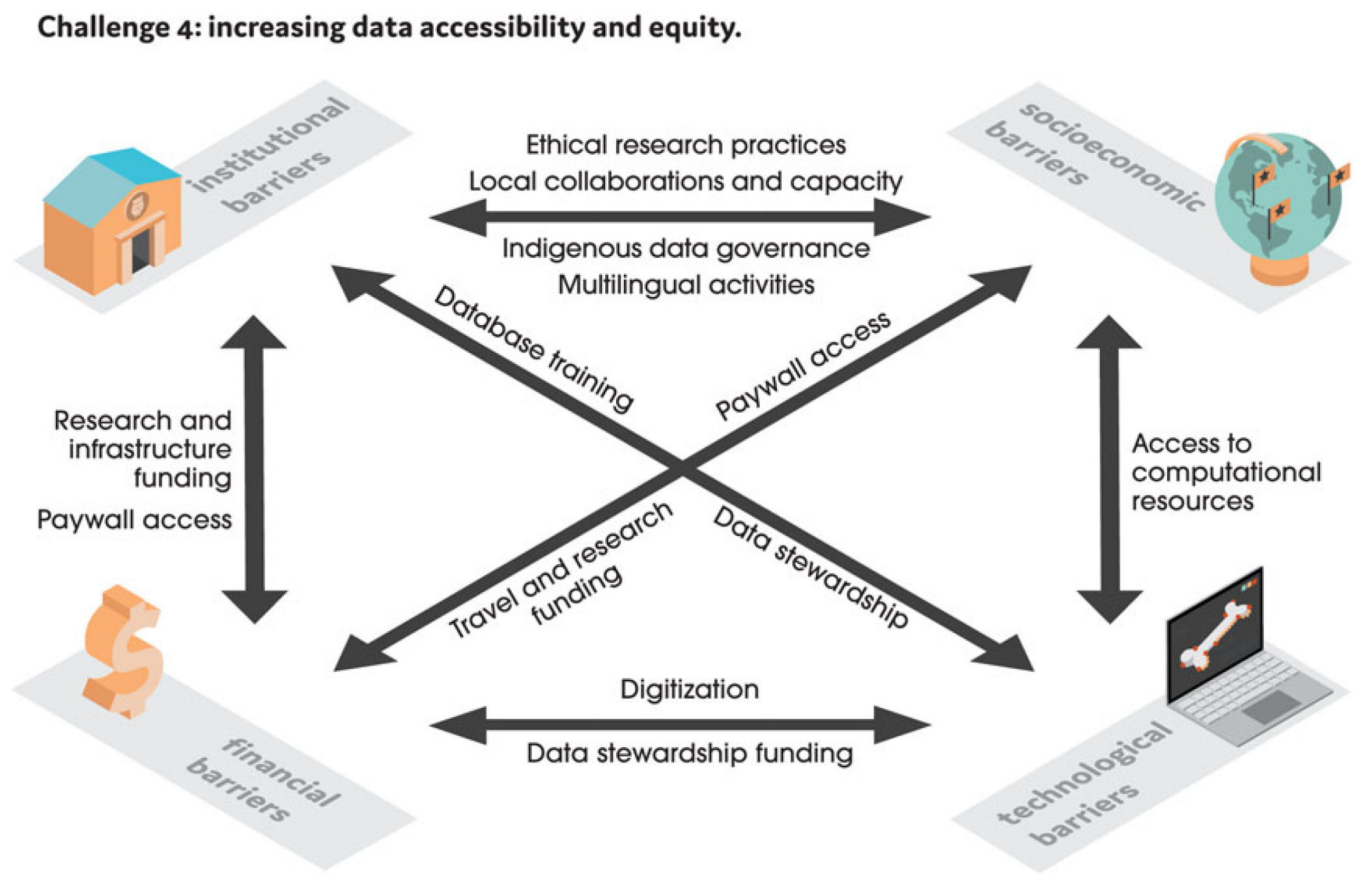
We identify four main barriers that hinder data accessibility and equity in analytical paleobiology: institutional (relating to museums, universities, and other research institutions), socioeconomic, technological, and financial. The arrows show relationships between these barriers and highlight where solutions are being applied.

Paleobiological data and computing resources are more accessible now than ever, but access to them is not equitable among researchers. Many financial, technological, institutional, and socioeconomic factors determine who participates in research as well as how paleobiological data are collected, interpreted, and shared (Núñez et al. 2020; Valenzuela-Toro and Viglino 2021) (Fig. 2). Advancing equity in the context of analytical paleobiology entails acknowledging that access to analytical resources is unequal and allocating them in relation to researchers’ needs to achieve fairer outcomes (CSSP 2019). Here, we discuss barriers pertaining to the access of paleobiological data and resources. These are by no means exhaustive but represent several broadscale challenges for which solutions have been proposed.

Fossil specimens and their associated morphological, geographic, and stratigraphic information underpin research in analytical paleobiology. Data collection often involves visiting museums or gathering digital data from publications and repositories. However, these data are not always accessible. Visiting museums to study specimens can be logistically, financially, or politically infeasible—or even impossible. Travel grants (e.g., John W. Wells Grants-in-Aid of Research Program at the Paleontological Research Institution) can help offset transportation costs, but they cannot alleviate visa issues or other travel restrictions. Likewise, data underlying publications might be buried in supplementary files or locked behind paywalls or might lack consistent metadata or formatting—if they are even made available. As such, emphasis could be placed on finding alternative ways to make paleobiological data more open, particularly for researchers who historically have had less access.

One major step forward is digitization. For example, many museums have committed to digitizing their collections (Nelson and Ellis 2019; Bakker et al. 2020; Hedrick et al. 2020; Sandramo et al. 2021). However, only a fraction of these “dark data” have been mobilized given the substantial time, money, and effort required (Nelson et al. 2012; Paterson et al. 2016; Marshall et al. 2018). If paleobiology continues to value digital data, financial and logistical support could be expanded for online databases and museum digitization efforts as well as resources for researchers to access those data.

Open-data practices do not end with digitization, however, as digital assets must also be maintained. In 2016, the FAIR Guiding Principles (Findability, Accessibility, Interoperability, and Reusability) for scientific data management and stewardship were published to enhance data discovery and reuse (Wilkinson et al. 2016). Additionally, the TRUST Principles (Transparency, Responsibility, User focus, Sustainability and Technology) were developed to demonstrate the trustworthiness of digital repositories (Lin et al. 2020). Although the biological sciences have embraced these principles, paleontology still lags behind (Stuart et al. 2018; Kinkade and Shepherd 2021). To encourage better data management practices, paleontological journals could require authors to archive their data, metadata, and code in centralized online repositories instead of only in supplementary files (Kaufman and PAGES 2k Special-Issue Editorial Team 2018). Unique dataset identifiers could, in turn, be adopted to track data reuse and credit the authors (Pierce et al. 2019). Normalizing these practices begins with data stewardship training to highlight resources (e.g., https://fairsharing.org) and community standards (e.g., Biodiversity Information Standards, https://www.tdwg.org) when managing paleobiological data (Koch et al. 2018; Seltmann et al. 2018; Stall et al. 2018; Krimmel et al. 2021).

As analytical paleobiology moves toward a future of open data, concerns regarding data ownership, representation, and control have been rekindled, particularly in relation to Indigenous communities and lands (Kukutai and Taylor 2016; Jennings et al. 2018; Rainie et al. 2019; McCartney et al. 2022). In response, the CARE Principles of Indigenous Data Governance (Collective Benefit, Authority to Control, Responsibility, and Ethics) were created to complement the FAIR Guiding Principles and promote the ethical use and reuse of Indigenous data (Carroll et al. 2020, 2021). Methods for implementing the FAIR Guiding Principles and CARE Principles in tandem (Rainie et al. 2019; Carroll et al. 2020, 2021) should be incorporated into analytical paleobiology courses to train researchers how to work with Indigenous data and partners without perpetuating entrenched power imbalances (Liboiron 2021; Monarrez et al. 2021).

Another dimension of access pertains to the language used to communicate information. Studies in analytical paleobiology rely heavily on information published in English (Raja et al. 2022). Although having a shared language of science can facilitate global collaboration, it also selectively excludes voices (Tardy 2004). For example, non-English publications are frequently omitted from data compilations, which might bias results from literature reviews (Amano et al. 2016, 2021; Nuñez and Amano 2021; Raja et al. 2022) and meta-analyses (Konno et al. 2020). To help alleviate language biases, researchers could conduct literature searches and disseminate their findings in multiple languages, advocate for translation or English proofing services at journals, and be considerate of non-native English speakers (Márquez and Porras 2020; Ramírez-Castañeda 2020; Amano et al. 2021; Gaynor et al. 2022; Steigerwald et al. 2022). Creating space for multilingual collaborations in analytical paleobiology would welcome knowledge, perspectives, and skills that might otherwise be overlooked due to language barriers.

Paleontology’ s history has left an indelible imprint on how research in the field is conducted today, contextualizing the challenges we highlight throughout this article. Knowledge production in analytical paleobiology, like other natural sciences, depends in part on socioeconomic factors such as wealth, education, and political stability, as well as colonial legacy (Boakes et al. 2010; Amano and Sutherland 2013; Hughes et al. 2021; Monarrez et al. 2021; Trisos et al. 2021; Raja et al. 2022). Consequently, sampling effort is not equally distributed across the world. For example, 97% of fossil occurrence data recorded in the Paleobiology Database over the last 30 years was generated by higher-income countries, particularly those in western Europe and North America (Raja et al. 2022). These socioeconomic factors intensify other geographic biases in the fossil record and warp biodiversity estimates (Challenge 1). As such, efforts to obtain a representative view of biodiversity across space and time are not disconnected from efforts to advance equity, inclusion, and ethics in analytical paleobiology. Recent publications have spotlighted actions that individuals and institutions should take to change research norms, urging our community to not only reflect on its past but forge a new path forward (Cronin et al. 2021; Liboiron 2021; Theodor et al. 2021; Cisneros et al. 2022; Dunne et al. 2022; Mohammed et al. 2022; Raja et al. 2022).

## Conclusion

Analytical paleobiology has grown in available data, computational power, and community interest over the last half century. Notably, progress in quantitative methods, conceptual frameworks, interdisciplinary partnerships, and data stewardship has contributed to more open and reproducible paleobiological research. These advances have expanded our ability to account for biases in the fossil record, accommodate different data types in models, integrate insights across disciplines, and pursue innovative research questions. Early-career researchers in particular, despite being precarious in terms of employment and career prospects, are embracing these evolving research practices. However, there is still a need to increase their acceptance among the broader paleontological community, establish best practices, and dismantle systemic inequities in how paleobiological data have historically been generated, shared, and accessed. Fortunately, we are not alone in facing these issues, and we can learn a great deal from solutions proposed by other disciplines. Great opportunity lies in both individual and institutional action to transform the future of how we study the past.

## Supplementary Material

Supplementary Materials

## Figures and Tables

**Table 1:** Summary of four challenges facing analytical paleobiology. Key advances are highlighted under each challenge.

**Challenge 1: Measuring biodiversity across space and time** Transparent reporting of taxonomic resolution and species-to-genus ratios
Taxonomy training and databases
Grants supporting systematics and curation Best practices and reproducible workflows for data standardization
Incentive structures that reward software development Training in quantitative methods
Awareness of sampling context and biases Interdisciplinary communication, collaboration, and funding
**Challenge 2: Integrating fossil and modern biodiversity data**
Data digitization and archiving using standardized protocols
Best practices and conceptual frameworks to guide data integration
Analyses that can accomodate heterogeneous datasets Development of taxon-free metrics
Training in quantitative methods
Interdisciplinary communication, collaboration, and funding
**Challenge 3: Building data science skills to analyze the fossil record**
Data science training
Collaboration with data scientists
**Challenge 4: Increasing data accessibility and equity** Travel support
Open data
Digitization resources
Data stewardship (FAIR Guiding Principles) Trustworthy digital repositories (TRUST Principles) Indigenous data governance (CARE Principles) Multilingual collaboration and database queries Local capacity building and research partnerships

## Data Availability

Supplementary Tables are available from the Zenodo Digital Repository: https://doi.org/10.5281/zenodo.7340036.

## References

[R1] Allen BJ, Wignall PB, Hill DJ, Saupe EE, Dunhill AM (2020). The latitudinal diversity gradient of tetrapods across the Permo-Triassic mass extinction and recovery interval. Proceedings of the Royal Society of London B.

[R2] Allmon WD (1992). Genera in paleontology: definition and significance. Historical Biology.

[R3] Allmon WD, Dietl GP, Hendricks JR, Ross RM, Rosenberg GD, Clary RM (2018). Museums at the forefront of the history and philosophy of geology: history made, history in the making.

[R4] Alroy J (2003). Global databases will yield reliable measures of global biodiversity. Paleobiology.

[R5] Alroy J (2010a). Fair sampling of taxonomic richness and unbiased estimation of origination and extinction rates. Paleontological Society Papers.

[R6] Alroy J (2010b). Geographical, environmental and intrinsic biotic controls on Phanerozoic marine diversification. Palaeontology.

[R7] Alroy J (2010c). The shifting balance of diversity among major marine animal groups. Science.

[R8] Alroy J (2020). On four measures of taxonomic richness. Paleobiology.

[R9] Alroy J, Aberhan M, Bottjer DJ, Foote M, Fürsich FT, Harries PJ, Hendy AJW, Holland SM, Ivany LC, Kiessling W, Kosnik MA (2008). Phanerozoic trends in the global diversity of marine invertebrates. Science.

[R10] Amano T, Sutherland WJ (2013). Four barriers to the global understanding of biodiversity conservation: wealth, language, geographical location and security. Proceedings of the Royal Society of London B.

[R11] Amano T, González-Varo JP, Sutherland WJ (2016). Languages are still a major barrier to global science. PLoS Biology.

[R12] Amano T, Rios Rojas C, Boum Y, Calvo M, Misra BB (2021). Ten tips for overcoming language barriers in science. Nature Human Behaviour.

[R13] Antell GS, Kiessling W, Aberhan M, Saupe EE (2020). Marine biodiversity and geographic distributions are independent on large scales. Current Biology.

[R14] Bakker FT, Antonelli A, Clarke JA, Cook JA, Edwards SV, Ericson PGP, Faurby S, Ferrand N, Gelang M, Gillespie RG, Irestedt M (2020). The Global Museum: natural history collections and the future of evolutionary science and public education. PeerJ.

[R15] Barido-Sottani J, Saupe EE, Smiley TM, Soul LC, Wright AM, Warnock RCM (2020). Seven rules for simulations in paleobiology. Paleobiology.

[R16] Beaufort L, Bolton CT, Sarr A-C, Suchéras-Marx B, Rosenthal Y, Donnadieu Y, Barbarin N, Bova S, Cornuault P, Gally Y, Gray E (2022). Cyclic evolution of phytoplankton forced by changes in tropical seasonality. Nature.

[R17] Benda LE, Poff LN, Tague C, Palmer MA, Pizzuto J, Cooper S, Stanley E, Moglen G (2002). How to avoid train wrecks when using science in environmental problem solving. BioScience.

[R18] Bennington JB, Dimichele WA, Badgley C, Bambach RK, Barrett PM, Behrensmeyer AK, Bobe R, Burnham RJ, Daeschler EB, Dam JV, Eronen JT (2009). Critical issues of scale in paleoecology. Palaios.

[R19] Benson RBJ, Butler R, Close RA, Saupe E, Rabosky DL (2021). Biodiversity across space and time in the fossil record. Current Biology.

[R20] Benton MJ (1995). Diversification and extinction in the history of life. Science.

[R21] Birks HJB, Lotter AF, Juggins S, Smol JP (2012). Tracking environmental change using lake sediments: data handling and numerical techniques.

[R22] Boakes EH, McGowan PJK, Fuller RA, Chang-qing D, Clark NE, O’Connor K, Mace GM (2010). Distorted views of biodiversity: spatial and temporal bias in species occurrence data. PLoS Biology.

[R23] Boulton AJ, Panizzon D, Prior J (2005). Explicit knowledge structures as a tool for overcoming obstacles to interdisciplinary research. Conservation Biology.

[R24] Brewer S, Jackson ST, Williams JW (2012). Paleoecoinfor-matics: applying geohistorical data to ecological questions. Trends in Ecology and Evolution.

[R25] Bryan J (2018). Excuse me, do you have a moment to talk about version control?. American Statistician.

[R26] Buma B, Harvey BJ, Gavin DG, Kelly R, Loboda T, McNeil BE, Marlon JR, Meddens AJH, Morris JL, Raffa KF, Shuman B (2019). The value of linking paleoecological and neoecological perspectives to understand spatially-explicit ecosystem resilience. Landscape Ecology.

[R27] Bush AM, Bambach RK (2004). Did alpha diversity increase during the Phanerozoic? Lifting the veils of taphonomic, latitudinal, and environmental biases. Journal of Geology.

[R28] Cantalapiedra J, Domingo LMS, Domingo L (2018). Multi-scale interplays of biotic and abiotic drivers shape mammalian sub-continental diversity over millions of years. Scientific Reports.

[R29] Carroll SR, Garba I, Figueroa-Rodríguez OL, Holbrook J, Lovett R, Materechera S, Parsons M, Raseroka K, Rodriguez-Lonebear D, Rowe R, Sara R (2020). The CARE Principles for Indigenous data governance. Data Science Journal.

[R30] Carroll SR, Herczog E, Hudson M, Russell K, Stall S (2021). Operationalizing the CARE and FAIR Principles for Indigenous data futures. Scientific Data.

[R31] [CSSP] Center for the Study of Social Policy (2019). Key equity terms and concepts: a glossary for shared understanding.

[R32] Chao A, Jost L (2012). Coverage-based rarefaction and extrapolation: standardizing samples by completeness rather than size. Ecology.

[R33] Chao A, Kubota Y, Zelený D, Chiu C-H, Li C-F, Kusumoto B, Yasuhara M, Thorn S, Wei C-L, Costello MJ, Colwell RK (2020). Quantifying sample completeness and comparing diversities among assemblages. Ecological Research.

[R34] Chao A, Henderson PA, Chiu C-H, Moyes F, Hu K-H, Dornelas M, Magurran AE (2021). Measuring temporal change in alpha diversity: a framework integrating taxonomic, phylogenetic and functional diversity and the iNEXT.3D standardization. Methods in Ecology and Evolution.

[R35] Chiarenza AA, Mannion PD, Lunt DJ, Farnsworth A, Jones LA, Kelland S-J, Allison PA (2019). Ecological niche modelling does not support climatically-driven dinosaur diversity decline before the Cretaceous/Paleogene mass extinction. Nature Communications.

[R36] Cisneros JC, Raja NB, Ghilardi AM, Dunne EM, Pinheiro FL, Regalado Fernández OR, Sales MAF, Rodríguez-de la Rosa RA, Miranda-Martínez AY, González-Mora S, Bantim RAM (2022). Digging deeper into colonial palaeontological practices in modern day Mexico and Brazil. Royal Society Open Science.

[R37] Cleary TJ, Benson RBJ, Evans SE, Barrett PM (2018). Lepidosaurian diversity in the Mesozoic-Palaeogene: the potential roles of sampling biases and environmental drivers. Royal Society Open Science.

[R38] Close RA, Benson RBJ, Upchurch P, Butler RJ (2017). Controlling for the species-area effect supports constrained long-term Mesozoic terrestrial vertebrate diversification. Nature Communications.

[R39] Close RA, Evers SW, Alroy J, Butler RJ (2018). How should we estimate diversity in the fossil record? Testing richness estimators using sampling-standardised discovery curves. Methods in Ecology and Evolution.

[R40] Close RA, Benson RBJ, Alroy J, Carrano MT, Cleary TJ, Dunne EM, Mannion PD, Uhen MD, Butler RJ (2020a). The apparent exponential radiation of Phanerozoic land vertebrates is an artefact of spatial sampling biases. Proceedings of the Royal Society of London B.

[R41] Close RA, Benson RBJ, Saupe EE, Clapham ME, Butler RJ (2020b). The spatial structure of Phanerozoic marine animal diversity. Science.

[R42] Condamine FL, Rolland J, Morlon H (2013). Macroevolution-ary perspectives to environmental change. Ecology Letters.

[R43] Costello MJ, Wilson S, Houlding B (2013). More taxonomists describing significantly fewer species per unit effort may indicate that most species have been discovered. Systematic Biology.

[R44] Cronin MR, Alonzo SH, Adamczak SK, Baker DN, Beltran RS, Borker AL, Favilla AB, Gatins R, Goetz LC, Hack N, Harenčár JG (2021). Anti-racist interventions to transform ecology, evolution and conservation biology departments. Nature Ecology and Evolution.

[R45] Davies AL, Streeter R, Lawson IT, Roucoux KH, Hiles W (2018). The application of resilience concepts in palaeoecology. The Holocene.

[R46] de Celis A, Narváez I, Arcucci A, Ortega F (2021). Lagerstätte effect drives notosuchian palaeodiversity (Crocodyliformes, Notosuchia). Historical Biology.

[R47] del Carmen Gomez Cabrera M, Young JM, Roff G, Staples T, Ortiz JC, Pandolfi JM, Cooper A (2019). Broadening the taxonomic scope of coral reef palaeoecological studies using ancient DNA. Molecular Ecology.

[R48] Dietl GP, Kidwell SM, Brenner M, Burney DA, Flessa KW, Jackson ST, Koch PL (2015). Conservation paleobiology: leveraging knowledge of the past to inform conservation and restoration. Annual Review of Earth and Planetary Sciences.

[R49] Dillon EM, McCauley DJ, Morales-Saldaña JM, Leonard ND, Zhao JX, O’Dea A (2021). Fossil dermal denticles reveal the preexploitation baseline of a Caribbean coral reef shark community. Proceedings of the National Academy of Sciences USA.

[R50] Doi H, Yasuhara M, Ushio M (2021). Causal analysis of the temperature impact on deep-sea biodiversity. Biology Letters.

[R51] Dunhill AM, Liow LH (2018). Crossing the palaeontological-ecological gap virtual issue. Methods in Ecology and Evolution.

[R52] Dunne EM, Raja NB, Stewens PP, Zin-Maung-Manug-Thein, Zaw K (2022). Ethics, law, and politics in palaeontological research: the case of Myanmar amber. Communications Biology.

[R53] Eigenbrode SD, O’Rourke M, Wulfhorst JD, Althoff DM, Goldberg CS, Merrill K, Morse W, Nielsen-Pincus M, Stephens J, Winowiecki L, Bosque-Pérez NA (2007). Employing philosophical dialogue in collaborative science. BioScience.

[R54] Eronen JT, Polly PD, Fred M, Damuth J, Frank DC, Mosbrugger V, Scheidegger C, Chr Stenseth N, Fortelius M (2010). Ecometrics: the traits that bind the past and present together. Integrative Zoology.

[R55] Ezard THG, Aze T, Pearson PN, Purvis A (2011). Interplay between changing climate and species’ ecology drives macroevo-lutionary dynamics. Science.

[R56] Fawcett S, Agosti D, Cole SR, Wright DF (2022). Digital accessible knowledge: mobilizing legacy data and the future of taxonomic publishing. Bulletin of the Society of Systematic Biologists.

[R57] Ferretti F, Crowder LB, Micheli F, Blight LK, Kittinger JN, McClenachan L, Gedan KB, Blight LK (2014). Marine historical ecology in conservation: applying the past to manage for the future.

[R58] Filazzola A, Lortie CJ (2022). A call for clean code to effectively communicate science. Methods in Ecology and Evolution.

[R59] Finnegan S, Anderson SC, Harnik PG, Simpson CD, Tittensor P, Byrnes JE, Finkel ZV, Lindberg DRL, Liow H, Lockwood R, Lotze HK (2015). Paleontological baselines for evaluating extinction risk in the modern oceans. Science.

[R60] Flannery-Sutherland JT, Silvestro D, Benton MJ (2022). Global diversity dynamics in the fossil record are regionally heterogeneous. Nature Communications.

[R61] Foote M (2000). Origination and extinction components of taxonomic diversity: general problems. Paleobiology.

[R62] Fraser D, Soul LC, Tóth AB, Balk MA, Eronen JT, Pineda-Munoz S, Shupinski AB, Villaseñor A, Barr WAA, Behrensmeyer K, Du A (2021). Investigating biotic interactions in deep time. Trends in Ecology and Evolution.

[R63] Gaynor KM, Azevedo T, Boyajian C, Brun J, Budden AE, Cole A, Csik S, DeCesaro J, Do-Linh H, Dudney J, Galaz García C (2022). Ten simple rules to cultivate belonging in collaborative data science research teams. PLoS Computational Biology.

[R64] Goodenough AE, Webb JC (2022). Learning from the past: opportunities for advancing ecological research and practice using palaeoecological data. Oecologia.

[R65] Gorneau JA, Ausich WI, Bertolino S, Bik H, Daly M, Demissew SD, Donoso A, Folk R, Freire-Fierro A, Ghazanfar SA, Grace OM (2022). Framing the future for taxonomic monography: improving recognition, support, and access. Bulletin of the Society of Systematic Biologists.

[R66] Goswami A, Watanabe A, Felice RN, Bardua C, Fabre A-C, Polly PD (2019). High-density morphometric analysis of shape and integration: the good, the bad, and the not-really-a-problem. Integrative and Comparative Biology.

[R67] Grenié M, Berti E, Carvajal-Quintero J, Dädlow GML, Sagouis A, Winter M (2023). Harmonizing taxon names in biodiversity data: a review of tools, databases and best practices. Methods in Ecology and Evolution.

[R68] Guerra CA, Heintz-Buschart A, Sikorski J, Chatzinotas A, Guerrero-Ramírez N, Cesarz S, Beaumelle L, Rillig MC, Maestre FT, Delgado-Baquerizo M, Buscot F (2020). Blind spots in global soil biodiversity and ecosystem function research. Nature Communications.

[R69] Guralnick RP, Hill AW, Lane M (2007). Towards a collaborative, global infrastructure for biodiversity assessment. Ecology Letters.

[R70] Hannisdal B, Liow LH (2018). Causality from palaeontological time series. Palaeontology.

[R71] Hart EM, Barmby P, LeBauer D, Michonneau F, Mount S, Mulrooney P, Poisot T, Woo KH, Zimmerman NB, Hollister JW (2016). Ten simple rules for digital data storage. PLoS Computational Biology.

[R72] Heath TA, Huelsenbeck JP, Stadler T (2014). The fossilized birth-death process for coherent calibration of divergence-time estimates. Proceedings of the National Academy of Sciences USA.

[R73] Heberling JM, Miller JT, Noesgaard D, Weingart SB, Schigel D (2021). Data integration enables global biodiversity synthesis. Proceedings of the National Academy of Sciences USA.

[R74] Hedrick BP, Heberling JM, Meineke EK, Turner KGC, Grassa J, Park DS, Kennedy J, Clarke JA, Cook JAD, Blackburn C, Edwards SV (2020). Digitization and the future of natural history collections. BioScience.

[R75] Hendricks JR, Saupe EE, Myers CE, Hermsen EJ, Allmon WD (2014). The generification of the fossil record. Paleobiology.

[R76] Hohmann N (2021). Incorporating information on varying sedimentation rates into paleontological analyses. Palaios.

[R77] Holland S, Loughney KM (2021). The stratigraphic paleobiology of nonmarine systems.

[R78] Hsiang AY, Nelson K, Elder LE, Sibert EC, Kahanamoku SS, Burke JE, Kelly A, Liu Y, Hull PM (2018). AutoMorph: accelerating morphometrics with automated 2D and 3D image processing and shape extraction. Methods in Ecology and Evolution.

[R79] Hsiang AY, Brombacher A, Rillo MC, Mleneck-Vautravers MJ, Conn S, Lordsmith S, Jentzen A, Henehan MJ, Metcalfe B, Fenton IS, Wade BS (2019). Endless forams: >34,000 modern planktonic foraminiferal images for taxonomic training and automated species recognition using convolutional neural networks. Paleoceanography and Paleoclimatology.

[R80] Huang H-HM, Yasuhara M, Horne DJ, Perrier V, Smith AJ, Brandão SN (2022). Ostracods in databases: state of the art, mobilization and future applications. Marine Micropaleontology.

[R81] Hughes AC, Orr MC, Ma K, Costello MJ, Waller J, Provoost P, Yang Q, Zhu C, Qiao H (2021). Sampling biases shape our view of the natural world. Ecography.

[R82] Jablonski D, Finarelli JA (2009). Congruence of morphologically-defined genera with molecular phylogenies. Proceedings of the National Academy of Sciences USA.

[R83] Jennings LL, David-Chavez DM, Martinez A, Lone Bear Rodriguez D, Rainie S (2018). Indigenous data sovereignty: How scientists and researchers can empower Indigenous data governance. American Geophysical Union, Fall Meeting.

[R84] Jones LA, Dean CD, Mannion PD, Farnsworth A, Allison PA (2021). Spatial sampling heterogeneity limits the detectability of deep time latitudinal biodiversity gradients. Proceedings of the Royal Society of London B.

[R85] Jones LA, Gearty W, Allen BJ, Eichenseer K, Dean CD, Galván S, Kouvari M, Godoy PL, Nicholl C, Buffan L, Dillon EM (2022). Palaeoverse: a community-driven R package to support palaeo-biological analyses. Earth ArXiv.

[R86] Karpatne A, Ebert-Uphoff I, Ravela S, Babaie HA, Kumar V (2019). Machine learning for the geosciences: challenges and opportunities. IEEE Transactions on Knowledge and Data Engineering.

[R87] Kaufman DS, PAGES 2k Special-Issue Editorial Team (2018). Technical note: open-paleo-data implementation pilot—the PAGES 2k special issue. Climate of the Past.

[R88] Kidwell SM (2015). Biology in the Anthropocene: challenges and insights from young fossil records. Proceedings of the National Academy of Sciences USA.

[R89] Kidwell SM, Holland SM (2002). The quality of the fossil record: implications for evolutionary analyses. Annual Review of Ecology and Systematics.

[R90] Kiessling W, Raja NB, Roden VJ, Turvey ST, Saupe EE (2019). Addressing priority questions of conservation science with palaeontological data. Philosophical Transactions of the Royal Society of London B.

[R91] Kinkade D, Shepherd A (2021). Geoscience data publication: practices and perspectives on enabling the FAIR guiding principles. Geoscience Data Journal.

[R92] Kliskey A, Alessa L, Wandersee S, Williams P, Trammell J, Powell J, Grunblatt J, Wipfli M (2017). A science of integration: frameworks, processes, and products in a place-based, integrative study. Sustainability Science.

[R93] Koch A, Glover KC, Zambri B, Thomas EK, Benito X, Yang JZ (2018). Open-data practices and challenges among early-career paleo-researchers. Past Global Change Magazine.

[R94] Kocsis ÁT, Reddin CJ, Alroy J, Kiessling W (2019). The r package divDyn for quantifying diversity dynamics using fossil sampling data. Methods in Ecology and Evolution.

[R95] König C, Weigelt P, Schrader J, Taylor A, Kattge J, Kreft H (2019). Biodiversity data integration—the significance of data resolution and domain. PLoS Biology.

[R96] Konno K, Akasaka M, Koshida C, Katayama N, Osada N, Spake R, Amano T (2020). Ignoring non-English-language studies may bias ecological meta-analyses. Ecology and Evolution.

[R97] Kopperud BT, Lidgard S, Liow LH (2019). Text-mined fossil biodiversity dynamics using machine learning. Proceedings of the Royal Society of London B.

[R98] Krimmel ET, Karim H, Little L, Walker R, Burkhalter C, Byrd A, Millhouse, Utrup J (2021). The Paleo Data Working Group: a model for developing and sustaining a community of practice. Biodiversity Information Science and Standards.

[R99] Kukutai T, Taylor J, Kukutai T, Taylor J (2016). Indigenous data sovereignty.

[R100] Labandeira CC, Sepkoski JJ (1993). Insect diversity in the fossil record. Science.

[R101] LaDeau SL, Han BA, Rosi-Marshall EJ, Weathers KC (2017). The next decade of big data in ecosystem science. Ecosystems.

[R102] Lendemer JC, Coyle JR (2021). Dissimilar biodiversity data sets yield congruent patterns and inference in lichens. Botany.

[R103] Levin SA (1992). The problem of pattern and scale in ecology: the Robert H. MacArthur award lecture. Ecology.

[R104] Lewandowska AM, Jonkers L, Auel H, Freund JA, Hagen W, Kucera M, Hillebrand H (2020). Scale dependence of temporal biodiversity change in modern and fossil marine plankton. Global Ecology and Biogeography.

[R105] Liboiron M (2021). Decolonizing geoscience requires more than equity and inclusion. Nature Geoscience.

[R106] Lin D, Crabtree J, Dillo I, Downs RR, Edmunds R, Giaretta D, De Giusti M, L’Hours H, Hugo W, Jenkyns R, Khodiyar V (2020). The TRUST Principles for digital repositories. Scientific Data.

[R107] Liow LH, Nichols JD (2010). Estimating rates and probabilities of origination and extinction using taxonomic occurrence data: capture-mark-recapture (CMR) approaches. Paleontological Society Papers.

[R108] Lowndes JSS, Best BD, Scarborough C, Afflerbach JC, Frazier MR, O’Hara CC, Jiang N, Halpern BS (2017). Our path to better science in less time using open data science tools. Nature Ecology and Evolution.

[R109] Lowndes JSS, Froehlich HE, Horst A, Jayasundara N, Pinsky ML, Stier AC, Therkildsen NO, Wood CL (2019). Supercharge your research: a ten-week plan for open data science. Nature.

[R110] Lyons KS, Amatangelo KL, Behrensmeyer AK, Bercovici A, Blois JL, Davis M, DiMichele WA, Du A, Eronen JT, Tyler Faith J, Graves GR (2016). Holocene shifts in the assembly of plant and animal communities implicate human impacts. Nature.

[R111] Márquez MC, Porras AM (2020). Science communication in multiple languages is critical to its effectiveness. Frontiers in Communication.

[R112] Marshall CR, Finnegan S, Clites EC, Holroyd PA, Bonuso N, Cortez C, Davis E, Dietl GP, Druckenmiller PS, Eng RC, Garcia C (2018). Quantifying the dark data in museum fossil collections as palaeontology undergoes a second digital revolution. Biology Letters.

[R113] Mathes GH, van Dijk J, Kiessling W, Steinbauer MJ (2021). Extinction risk controlled by interaction of long-term and short-term climate change. Nature Ecology and Evolution.

[R114] McCartney AM, Anderson J, Liggins L, Hudson ML, Anderson MZ, TeAika B, Geary J, Cook-Deegan R, Patel HR, Phillippy AM (2022). Balancing openness with Indigenous data sovereignty: an opportunity to leave no one behind in the journey to sequence all of life. Proceedings of the National Academy of Sciences USA.

[R115] McClenachan L, Cooper AB, McKenzie MG, Drew JA (2015). The importance of surprising results and best practices in historical ecology. BioScience.

[R116] McKay NP, Emile-Geay J, Khider D (2021). geoChronR—an R package to model, analyze, and visualize age-uncertain data. Geochronology.

[R117] McKinney ML, Drake JA (2001). Biodiversity dynamics: turnover of populations, taxa, and communities.

[R118] Michener WK (2015). Ten simple rules for creating a good data management plan. PLoS Computational Biology.

[R119] Mohammed RS, Turner G, Fowler K, Pateman M, Nieves-Colón MA, Fanovich L, Cooke SB, Dávalos LM, Fitzpatrick SM, Giovas CM, Stokowski M (2022). Colonial legacies influence biodiversity lessons: how past trade routes and power dynamics shape present-day scientific research and professional opportunities for Caribbean scientists. American Naturalist.

[R120] Monarrez PM, Zimmt JB, Clement AM, Gearty W, Jacisin JJK, Jenkins M, Kusnerik KM, Poust AW, Robson SV, Sclafani JA, Stilson KT (2021). Our past creates our present: a brief overview of racism and colonialism in Western paleontology. Paleobiology.

[R121] Morrison SA, Sillett TS, Funk WC, Ghalambor CK, Rick TC (2017). Equipping the 22nd-century historical ecologist. Trends in Ecology and Evolution.

[R122] Mottl O, Grytnes J-A, Seddon AWR, Steinbauer MJK, Bhatta P, Felde VA, Flantua SGA, Birks HJB (2021). Rate-of-change analysis in paleoecology revisited: a new approach. Review of Palaeobotany and Palynology.

[R123] Moudrý V, Devillers R (2020). Quality and usability challenges of global marine biodiversity databases: an example for marine mammal data. Ecological Informatics.

[R124] Mouillot D, Graham NAJ, Villéger S, Mason NWH, Bellwood DR (2013). A functional approach reveals community responses to disturbances. Trends in Ecology and Evolution.

[R125] Muñoz MM, Price SA (2019). The future is bright for evolutionary morphology and biomechanics in the era of big data. Integrative and Comparative Biology.

[R126] Muscente AD, Prabhu A, Zhong H, Eleish A, Meyer MB, Fox P, Hazen RM, Knoll AH (2018). Quantifying ecological impacts of mass extinctions with network analysis of fossil communities. Proceedings of the National Academy of Sciences USA.

[R127] Napier JD, Chipman ML (2022). Emerging palaeoecological frameworks for elucidating plant dynamics in response to fire and other disturbance. Global Ecology and Biogeography.

[R128] Nelson G, Ellis S (2019). The history and impact of digitization and digital data mobilization on biodiversity research. Philosophical Transactions of the Royal Society of London B.

[R129] Nelson G, Paul D, Riccardi G, Mast A (2012). Five task clusters that enable efficient and effective digitization of biological collections. ZooKeys.

[R130] Nieto-Lugilde D, Blois JL, Bonet-García FJ, Giesecke T, Gil-Romera G, Seddon A (2021). Time to better integrate paleoecological research infrastructures with neoecology to improve understanding of biodiversity long-term dynamics and to inform future conservation. Environmental Research Letters.

[R131] Nosek BA, Alter G, Banks GC, Borsboom D, Bowman SD, Breckler SJ, Buck S, Chambers CD, Chin G, Christensen G, Contestabile M (2015). Promoting an open research culture. Science.

[R132] Núñez A-M, Rivera J, Hallmark T (2020). Applying an inter-sectionality lens to expand equity in the geosciences. Journal of Geoscience Education.

[R133] Nuñez MA, Amano T (2021). Monolingual searches can limit and bias results in global literature reviews. Nature Ecology and Evolution.

[R134] Olsen AM, Westneat MW (2015). StereoMorph: an Rpackage for the collection of 3D landmarks and curves using a stereo camera set-up. Methods in Ecology and Evolution.

[R135] Paterson G, Albuquerque S, Blagoderov V, Brooks S, Cafferty S, Cane E, Carter V, Chainey J, Crowther R, Douglas L, Durant J (2016). iCollections—digitising the British and Irish butterflies in the Natural History Museum, London. Biodiversity Data Journal.

[R136] Patzkowsky ME, Holland SM (2012). Stratigraphic paleobiology: understanding the distribution of fossil taxa in time and space.

[R137] Peters SE, Zhang C, Livny M, Ré C (2014). A machine reading system for assembling synthetic paleontological databases. PLoS ONE.

[R138] Pierce HH, Dev A, Statham E, Bierer BE (2019). Credit data generators for data reuse. Nature.

[R139] Pimiento C, Griffin JN, Clements CF, Silvestro D, Varela S, Uhen MD, Jaramillo C (2017). The Pliocene marine megafauna extinction and its impact on functional diversity. Nature Ecology and Evolution.

[R140] Pimiento C, Leprieur F, Silvestro D, Lefcheck JS, Albouy C, Rasher DB, Davis M, Svenning J-C, Griffin JN (2020). Functional diversity of marine megafauna in the Anthropocene. Science Advances.

[R141] Pinzón JH, Sampayo E, Cox E, Chauka LJ, Chen CA, Voolstra CR, LaJeunesse TC (2013). Blind to morphology: genetics identifies several widespread ecologically common species and few endemics among Indo-Pacific cauliflower corals (Pocillopora, Scleractinia). Journal of Biogeography.

[R142] Price SA, Schmitz L (2016). A promising future for integrative biodiversity research: an increased role of scale-dependency and functional biology. Philosophical Transactions of the Royal Society of London B.

[R143] Purnell MA, Donoghue PJC, Gabbott SE, McNamara ME, Murdock DJE, Sansom RS (2018). Experimental analysis of soft-tissue fossilization: opening the black box. Palaeontology.

[R144] Rainie S, Kukutai T, Walter M, Figueroa-Rodriguez O, Walker J, Axelsson P, Davies T, Walker S, Rubinstein M, Perini F (2019). The state of open data: histories and horizons.

[R145] Raja NB, Lauchstedt A, Pandolfi JM, Kim SW, Budd AF, Kiessling W (2021). Morphological traits of reef corals predict extinction risk but not conservation status. Global Ecology and Biogeography.

[R146] Raja NB, Dunne EM, Matiwane A, Khan TM, Nätscher PS, Ghilardi AM, Chattopadhyay D (2022). Colonial history and global economics distort our understanding of deep-time biodiversity. Nature Ecology and Evolution.

[R147] Ramírez-Castañeda V (2020). Disadvantages in preparing and publishing scientific papers caused by the dominance of the English language in science: the case of Colombian researchers in biological sciences. PLoS ONE.

[R148] Rapacciuolo G, Blois JL (2019). Understanding ecological change across large spatial, temporal and taxonomic scales: integrating data and methods in light of theory. Ecography.

[R149] Raup DM (1972). Taxonomic diversity during the Phanerozoic. Science.

[R150] Raup DM (1976). Species diversity in the Phanerozoic: an interpretation. Paleobiology.

[R151] Raup DM (1991). The future of analytical paleobiology. Short Courses in Paleontology.

[R152] Raup DM, Sepkoski JJ (1982). Mass extinctions in the marine fossil record. Science.

[R153] Raup DM, Gould SJ, Schopf TJM, Simberloff DS (1973). Stochastic models of phylogeny and the evolution of diversity. Journal of Geology.

[R154] Roswell M, Dushoff J, Winfree R (2021). A conceptual guide to measuring species diversity. Oikos.

[R155] Rull V (2010). Ecology and palaeoecology: two approaches, one objective. Open Ecology Journal.

[R156] Runge J, Bathiany S, Bollt E, Camps-Valls G, Coumou D, Deyle E, Glymour C, Kretschmer M, Mahecha MD, Muñoz-Marí J, van Nes EH (2019). Inferring causation from time series in Earth system sciences. Nature Communications.

[R157] Sandramo D, Nicosia E, Cianciullo S, Muatinte B, Guissamulo A (2021). Unlocking the entomological collection of the natural history museum of Maputo, Mozambique. Biodiversity Data Journal.

[R158] Sandve GK, Nekrutenko A, Taylor J, Hovig E (2013). Ten simple rules for reproducible computational research. PLoS Computational Biology.

[R159] Scarponi D, Nawrot R, Azzarone M, Pellegrini C, Gamberi F, Trincardi F, Kowalewski M (2022). Resilient biotic response to long-term climate change in the Adriatic Sea. Global Change Biology.

[R160] Schoon M, van der Leeuw S (2015). The shift toward social-ecological systems perspectives: insights into the humannature relationship. Natures Sciences Sociétés.

[R161] Seddon AWR, Mackay AW, Baker AG, Birks HJB, Breman E, Buck CE, Ellis EC, Froyd CA, Gill JL, Gillson L, Johnson EA (2014). Looking forward through the past: identification of 50 priority research questions in palaeoecology. Journal of Ecology.

[R162] Seltmann K, Lafia S, Paul D, James S, Bloom D, Rios N, Ellis S, Farrell U, Utrup J, Yost M, Davis E (2018). Georeferencing for Research Use (GRU): an integrated geospatial training paradigm for biocollections researchers and data providers. Research Ideas and Outcomes.

[R163] Sepkoski JJ (1981). A factor analytic description of the Phanerozoic marine fossil record. Paleobiology.

[R164] Sepkoski JJ (1997). Biodiversity: past, present, and future. Journal of Paleontology.

[R165] Sepkoski JJ, Bambach RK, Raup DM, Valentine JW (1981). Phanerozoic marine diversity and the fossil record. Nature.

[R166] Shaw JO, Briggs DEG, Hull PM (2020). Fossilization potential of marine assemblages and environments. Geology.

[R167] Sievanen L, Campbell LM, Leslie HM (2012). Challenges to interdisciplinary research in ecosystem-based management. Conservation Biology.

[R168] Signor PW, Gilinsky NL (1991). Introduction to analytical paleobiology. Short Courses in Paleontology.

[R169] Silvestro D, Salamin N, Schnitzler J (2014). PyRate: a new program to estimate speciation and extinction rates from incomplete fossil data. Methods in Ecology and Evolution.

[R170] Simpson GL (2018). Modelling palaeoecological time series using generalised additive models. Frontiers in Ecology and Evolution.

[R171] Smith AB, McGowan AJ (2011). The ties linking rock andfos-sil records and why they are important for palaeobiodiversity studies. Geological Society of London Special Publication.

[R172] Spalding C, Hull PM (2021). Towards quantifying the mass extinction debt of the Anthropocene. Proceedings of the Royal Society of London B.

[R173] Stall S, Yarmey LR, Boehm R, Cousijn H, Cruse P, Cutcher-Gershenfeld J, Dasler R, de Waard A, Duerr R, Elger K, Fenner M (2018). Advancing FAIR data in earth, space, and environmental science. Eos.

[R174] Steigerwald E, Ramírez-Castañeda V, Brandt DYC, Báldi A, Shapiro JT, Bowker L, Tarvin RD (2022). Overcoming language barriers in academia: machine translation tools and a vision for a multilingual future. BioScience.

[R175] Stuart D, Baynes G, Hrynaszkiewicz I, Allin K, Penny D, Lucraft M, Astell M (2018). Practical challenges for researchers in data sharing.

[R176] Su DF, Croft DA, Croft DA, Su DF, Simpson SW (2018). Methods in paleoecology: reconstructing Cenozoic terrestrial environments and ecological communities.

[R177] Szabó P, Hédl R (2011). Advancing the integration of history and ecology for conservation. Conservation Biology.

[R178] Tardy C (2004). The role of English in scientific communication: *lingua franca or Tyrannosaurus rex*?. Journal of English for Academic Purposes.

[R179] Theodor JMM, Lewis E, Rayfield EJ (2021). Amber specimens acquired from Myanmar following military coup.

[R180] Tomašových A, Kidwell SM (2010). Predicting the effects of increasing temporal scale on species composition, diversity, and rank-abundance distributions. Paleobiology.

[R181] Tomašových A, Kidwell SM, Barber RF (2016). Inferring skeletal production from time-averaged assemblages: skeletal loss pulls the timing of production pulses towards the modern period. Paleobiology.

[R182] Tomašových A, Albano PG, Fuksi T, Gallmetzer I, Haselmair A, Kowalewski M, Nawrot R, Nerlović V, Scarponi D, Zuschin M (2020). Ecological regime shift preserved in the Anthropocene stratigraphic record. Proceedings of the Royal Society of London B.

[R183] Trisos CH, Auerbach J, Katti M (2021). Decoloniality and anti-oppressive practices for a more ethical ecology. Nature Ecology and Evolution.

[R184] Uhen MD, Buckland PI, Goring SJ, Jenkins JP, Williams JW (2021). The EarthLife Consortium API: an extensible, open-source service for accessing fossil data and taxonomies from multiple community paleodata resources. Frontiers of Biogeography.

[R185] Valentine JW (1969). Patterns of taxonomic and ecological structure of the shelf benthos during Phanerozoic time. Palaeontology.

[R186] Valenzuela-Toro AM, Viglino M (2021). How Latin American researchers suffer in science. Nature.

[R187] Varela S, González-Hernández J, Sgarbi LF, Marshall C, Uhen MD, Peters S, McClennen M (2015). paleobioDB: an R package for downloading, visualizing and processing data from the Paleobiology Database. Ecography.

[R188] Vellend M, Brown CD, Kharouba HM, McCune JL, Myers-Smith IH (2013). Historical ecology: using unconventional data sources to test for effects of global environmental change. American Journal of Botany.

[R189] Vilhena DA, Smith AB (2013). Spatial bias in the marine fossil record. PLoS ONE.

[R190] Walker FM, Dunhill AM, Benton MJ (2020). Variable preservation potential and richness in the fossil record of vertebrates. Palaeontology.

[R191] Warnock RCM, Heath TA, Stadler T (2020). Assessing the impact of incomplete species sampling on estimates of speciation and extinction rates. Paleobiology.

[R192] Wilke T, Wagner B, Van Bocxlaer B, Albrecht C, Ariztegui D, Delicado D, Francke A, Harzhauser M, Hauffe T, Holtvoeth J, Just J (2016). Scientific drilling projects in ancient lakes: integrating geological and biological histories. Global and Planetary Change.

[R193] Wilkinson MD, Dumontier M, Aalbersberg IJ, Appleton G, Axton M, Baak A, Blomberg N, Boiten J-W, da Silva Santos LB, Bourne PE, Bouwman J (2016). The FAIR Guiding Principles for scientific data management and stewardship. Scientific Data.

[R194] Willis KJ, Birks HJB (2006). What is natural? The need for a long-term perspective in biodiversity conservation. Science.

[R195] Wilson G, Bryan J, Cranston K, Kitzes J, Nederbragt L, Teal TK (2017). Good enough practices in scientific computing. PLoS Computational Biology.

[R196] Wolkovich EM, Cook BI, McLauchlan KK, Davies TJ (2014). Temporal ecology in the Anthropocene. Ecology Letters.

[R197] Womack TM, Crampton JS, Hannah MJ (2021). Spatial scaling of beta diversity in the shallow-marine fossil record. Paleobiology.

[R198] Wüest RO, Zimmermann NE, Zurell D, Alexander JM, Fritz SA, Hof C, Kreft H, Normand S, Cabral JS, Szekely E, Thuiller W (2020). Macroecology in the age of big data—where to go from here?. Journal of Biogeography.

[R199] Yasuhara M, Doi H, Wei C-L, Danovaro R, Myhre SE (2016). Biodiversity-ecosystem functioning relationships in long-term time series and palaeoecological records: deep sea as a test bed. Philosophical Transactions of the Royal Society of London B.

[R200] Yasuhara M, Tittensor DP, Hillebrand H, Worm B (2017). Combining marine macroecology and palaeoecology in understanding biodiversity: microfossils as a model. Biological Reviews.

[R201] Yasuhara M, Huang H-H, Hull P, Rillo M, Condamine F, Tittensor D, Kučera M, Costello M, Finnegan S, O’Dea A, Hong Y (2020). Time machine biology: cross-timescale integration of ecology, evolution, and oceanography. Oceanography.

[R202] Zamani A, Fric ZF, Gante HF, Hopkins T, Orfinger AB, Scherz MD, Bartoňová AS, Pos DD (2022). DNA barcodes on their own are not enough to describe a species. Systematic Entomology.

[R203] Zeppelini D, Dal Molin A, Lamas CJE, Sarmiento C, Rheims CA, Fernandes DRR, Lima EFB, Silva EN, Carvalho-Filho F, Montoya-Lerma J, Moldovan OT (2021). The dilemma of self-citation in taxonomy. Nature Ecology and Evolution.

